# *Family Talk* versus usual services in improving child and family psychosocial functioning in families with parental mental illness: a randomised controlled trial and cost analysis

**DOI:** 10.3389/fpsyt.2024.1287378

**Published:** 2024-03-28

**Authors:** Mairead Furlong, Colm McGuinness, Christine Marie Mulligan, Sharon Lisa McGarr, Sinead McGilloway

**Affiliations:** ^1^ Centre for Mental Health and Community Research, Maynooth University Department of Psychology and Social Sciences Institute, Maynooth, Ireland; ^2^ Technological University Dublin, Dublin, Ireland

**Keywords:** children, COPMI, Family Talk, family-focused practice, mental health, mental disorder, parents, randomised controlled trial

## Abstract

**Background:**

Parental mental illness (PMI) is common and places children at high risk of developing psychological disorders. *Family Talk* (FT) is a well-known, whole-family, 7-session intervention designed to reduce the risk of transgenerational psychopathology. However, very few larger-scale evaluations of FT (across only a limited number of settings) have been conducted to date while there have been no cost analyses. This study aimed to assess the effectiveness and costs of delivering FT in improving child and family psychosocial functioning in families with PMI within routine mental health settings.

**Methods:**

A total of 83 families with PMI, with children aged 5-18 years, were randomly assigned on a 2:1 ratio to receive either the FT intervention (n=55 families) or usual services (n=28 families) across 10 adult, child and primary care mental health sites in Ireland. Parental disorders included anxiety/depression (57%), Bipolar Disorder (20%), Borderline Personality Disorder (12%), Post-Traumatic Stress Disorder (8%) and psychosis (2%). Detailed assessments with parents were conducted at baseline and 6-month follow up.

**Results:**

FT led to significant improvements in family functioning and child behaviour at 6-month follow up when compared to usual services, with medium effect sizes indicated. Parent participants with lower mental health literacy at baseline also showed significant post-intervention improvements. Those parents with less severe mental illness at baseline, and families with more partner and economic supports, reported additional significant post-intervention improvements in child depression/anxiety and parental mental health symptoms. The cost of FT amounted to €761.50 per family, although this decreased to €415.31 when recurring costs only were included.

**Conclusion:**

The findings from this study, which was conducted within the context of a national programme to introduce family-focused practice in Ireland, demonstrate that FT is a low-cost intervention that improved child and family psychosocial functioning across different mental health disorders within routine adult, child and primary care mental health services. The findings contribute to the growing evidence base for FT, and provide a robust basis to inform practice and policy development for families with parental mental illness both in Ireland and elsewhere.

**Clinical trial registration:**

https://www.isrctn.com/ISRCTN13365858, identifier ISRCTN13365858.

## Introduction

It is estimated that almost one in four children (23%) has a parent with mental illness (PMI) (1). These children present a 41%–77% lifetime risk of developing serious mental illness and impaired psychosocial outcomes and are five times more likely to use health and social services (2, 3). Traditionally, both in Ireland and in other jurisdictions, these families have remained invisible and unsupported due to a number of factors including: the segregation of adult and child mental health services; an individualised, crisis-oriented approach to assessment/treatment; competency and confidentiality concerns amongst mental health professionals; and parental stigma/fear of social services and losing custody of their children (4, 5).

Given the prevalence and burden of PMI, there has been a growing recognition in many countries of the need to support families in order to protect children from developing mental health disorders (6). Reassuringly, a range of interventions has been developed (e.g. targeting parents, children, or whole family), with collective evidence that they can decrease the risk of developing mental disorders for children by up to 40% (6, 7). In particular, whole-family programmes that include *both* parents and young people have been found to be more effective in reducing child psychopathology and referrals to child protection services, and in improving family relationships, than those which focus on either group alone (8–10). Such interventions allow multiple, often hidden concerns and burdens to be revealed and shared (from the perspectives of the parent with mental illness, partner, and children), thereby reducing stigma and guilt, and enhancing mutual understanding, support, problem-solving, care-planning and family relationships (9–12). Whole-family approaches also encourage typically fragmented adult and child mental health services to work together to support these often invisible children (5, 13).

**Figure 1:**
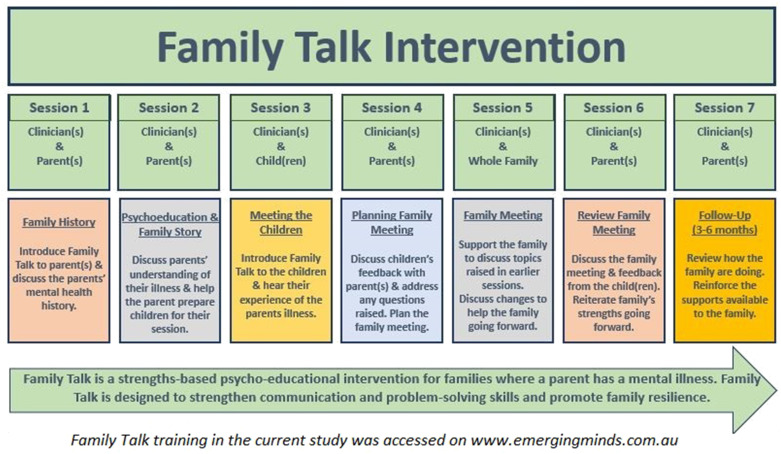
Family Talk sessions.

Whole-family programmes may be delivered across a range of settings (inpatient, outpatient, home) and vary in content (e.g. psycho-education, cognitive-behavioural therapy and/or parenting skills), duration (from a single session up to 18 months) and severity of PMI (14–17). Family Talk (FT), in particular, has emerged as a promising intervention due to its evidence base, duration and provision of low-cost, high quality training/manualised materials (5, 14). FT is a whole-family, 7-session, manualised, clinician-facilitated programme based on psychoeducation, systemic therapy, and a strengths-based narrative approach designed to improve family understanding and communication about parental mental illness, reduce stigma, and promote problem solving, family resilience, relationships and social supports (18–22). As part of the programme, a practitioner meets with each individual family, that is, with the parents (sessions 1, 2, 6, 7), with each child individually (session 3), and with the whole family together (session 4) (**Figure 1**). Evidence indicates that at post-intervention and 1.5 year follow up, FT improves child internalising and externalising symptoms and improves family understanding of mental illness (14, 18–21, 23–26). There is also growing evidence that FT may enhance family functioning and parenting self-efficacy, while reducing parental mental health symptoms (21, 24, 26). For example, one study indicated that diagnoses of parental affective and non-affective disorders decreased respectively from 90% to 66% and from 43% to 31% at 4.5year follow up (21). FT has been implemented in recent years in several countries as part of national initiatives to support families where a parent has mental illness(e.g., the USA, Costa Rica, Colombia, the Netherlands, Greece, Scandinavia, Iceland, and Australia) (19).

However, the evidence base for FT is in need of further development, for a number of reasons. Firstly, only two previous studies have compared FT to a treatment-as-usual/waitlist control group, both of which were non-randomised (23, 26), while the remaining randomised controlled trials (RCTs) compared FT to an active intervention (14, 18, 20, 23–26), which can make it difficult to assert a treatment or prevention effect. Secondly, some of the sample sizes in these studies were small (e.g. three were<40) and conducted within only a limited number of settings (e.g. USA, Finland, Germany, Greece) (14, 18, 20, 23–26). Thirdly, three of the RCTs were evaluated by the programme developer within a controlled ‘efficacy trial’ setting (14, 18, 20), thereby highlighting a need for more independent effectiveness trials of FT conducted within routine service settings and across different cultural contexts. Fourthly, evidence for the effectiveness of FT in improving child psychological functioning is mixed, with three studies indicating no improvements in parent report of child internalising and/or externalising symptoms (21, 23, 27). Moreover, given that enhanced family communication and functioning is a key objective of FT (14), further investigation of this important outcome is required within effectiveness studies.

In addition, FT has been primarily evaluated, to date, with parents with depression, largely because it was originally developed for this target group (19). Nevertheless, FT has been more widely implemented in some jurisdictions (e.g. Sweden, Finland) with parents with a range of mental disorders (28–30). Qualitative analyses conducted in Sweden reported that FT is safe and acceptable for parents with anxiety, Attention Deficit Hyperactivity Disorder, anxiety, Bipolar Disorder, depression, Personality Disorder, Post-Traumatic Stress Disorder, psychosis and substance abuse (22, 28–31). Interestingly, the two non-randomised studies, conducted in Germany and Sweden, included parents with depression, anxiety, attention deficit hyperactivity disorder, and bipolar disorder and reported largely positive results (23, 26). Therefore, there is a need for more high quality RCTs to assess FT in order to enhance the evidence base for its use with a wider range of mental health disorders. Furthermore, we know little about which populations may benefit most from FT. Longitudinal studies indicate that variables such as severity of parental mental illness, socioeconomic status, and availability of familial and service supports may moderate the risk of children with PMI developing a mental disorder (2, 32). Similarly, attritional analyses from recent quantitative and qualitative studies of FT also suggest that socioeconomic status and/or severity of PMI may influence intervention effectiveness (22, 24). Lastly, costs are a critical consideration for governments when allocating funding and resources (6) but there is an absence of costs analyses in informing the implementation of FT, and indeed family-focused practice more generally, within routine mental health settings.

Ireland lags behind most European countries and Australia as it does not have a legislative framework and/or a national “think family” policy/practice guidance to identify and support families with PMI (15, 33). Consequently, the funding provided by the national Health Service Executive for the current ‘PRIMERA’ research (Promoting Research and Innovation in Mental hEalth seRvices for fAmilies and children) was crucial in supporting the first endeavour to systematically implement family-focused practice (FFP) for families with PMI in Ireland. The overarching aims of the multi-strand PRIMERA project were to identify/develop, implement, and evaluate a family-focused intervention for families with PMI and, by so doing, help to inform a “think family” care delivery agenda within mental health services in Ireland. The project involved three key phases including: (1) an initial scoping study (including literature review and installation phase) to inform the identification and implementation of an appropriate programme in Ireland (in this case FT) *(Phase 1*); (2) an RCT and cost analysis to examine impact of the programme and some initial costings *(Phase 2);* and (3) a process evaluation to explore the theory of change and key contextual factors that affect the implementation and effectiveness of FT *(Phase 3)* (24). This paper focuses only on Phase 2. The findings from Phases 1 and 3 are reported elsewhere (5, 11, 12).

In the first phase of this research (2017–2018), we conducted a scoping study of FFP across adult (*n* = 114) and child (*n* = 69) mental health services in Ireland, and found that support for families was either non-existent, in the planning stages, or *ad hoc* and small scale (5). It was subsequently decided on the basis of a review of the literature and in conjunction with stakeholders, to implement FT. Findings from the stakeholder consultation (involving funders, service providers and service users) indicate that FT was selected because it: encourages collaboration between traditionally segregated adult and child mental health services in supporting parental mental illness; is a manualised, evidence-based, ‘whole family’ approach that works with both parents and children; has high quality, freely available, online training/resources; and was considered to be replicable and capable of being implemented across sites in Ireland (5). The specific objectives of the study reported here *(Phase 2)* were to conduct a randomised controlled trial and a costs analysis to assess the effectiveness (and costs) of FT when compared to usual services, in improving child and family psychosocial functioning in families with PMI in Ireland.

## Methods

### Participants and settings

FT was installed and implemented in 15 sites in Ireland between 2017and 2021. Ten of the 15 FT sites recruited eligible families for the trial, including five Adult Mental Health Services (AMHS), one Child and Adolescent Mental Health Service (CAMHS), one site affiliated to the statutory child welfare and protection agency in Ireland (called ‘Tusla’), and four interagency networks involving liaison among AMHS, CAMHS, Tusla and primary care services. The interagency networks involved a local champion establishing a working group for family-focused practice in the region, which coordinated liaison, referrals, co-working and supervision across clinicians in adult and child services in the area in engaging families for FT and the research. (Five sites did not include families in the trial due to ineligibility of recruited participants, staff deficits and/or the impact of COVID-19 restrictions.). FT was delivered within community outpatient clinics in both rural and urban areas, with a minority (< 15%) taking place in the home setting. Families were eligible for inclusion in the study if parent(s) were aged over 18, had children aged 5–18 years, and were either (a) attending AMHS due to a formal (or working) diagnosis of mental illness, and under the care of a consultant psychiatrist/multi-disciplinary team; or (b) attending their general practitioner (GP) for mental illness. The stability of the parents’ symptoms was ascertained by the clinical practitioner in conjunction with the parent and a joint decision reached as to their ability to engage with the intervention and the research. Family Talk sessions were postponed/stopped if clinically required (e.g. due to a relapse in parental mental health symptoms), or if requested by the family (e.g. family crisis takes priority).

**Figure 2:**
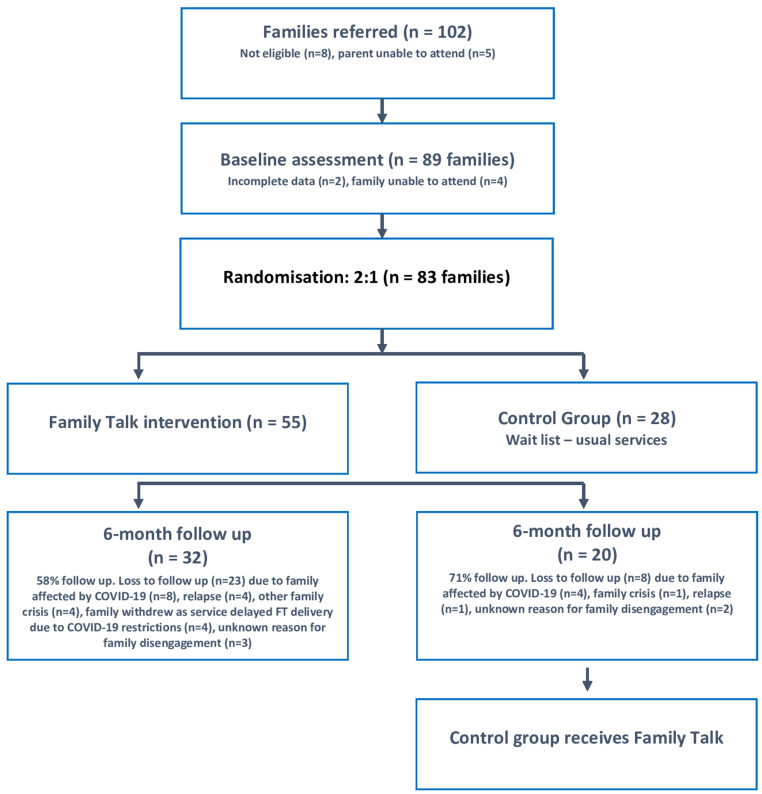
Study flow diagram.

Due to the high risk of intergenerational transmission of mental disorders (2), and a desire among stakeholders to increase family-focused collaboration between traditionally segregated AMHS and CAMHS (5), we included families where children attended CAMHS or primary care services for mental health issues, as well as families where children were not involved with mental health services. Families were excluded if parents or family members were unable to engage due to, for instance, active psychosis, substance misuse, custody dispute, urgent need for child protection services, or parent/child in hospital. In total, following baseline assessments and randomisation, 83 families were eligible to participate in the research. It should be noted, however, that the recruitment of new families was severely impacted by the COVID-19 public safety restrictions introduced in 2020-2021 in Ireland (recruitment commenced in March 2019 and was extended to April 2021 due to the lockdowns, but only 9 new families were recruited after the onset of COVID-19 in March 2020). At 6-month follow up (T1), we obtained data for 52 families, which represented 37% attrition, the rate of which doubled due to the impact of COVID-19 restrictions (23% vs. 45%). For the same reason, it was not possible to conduct the planned 12-month follow up (T2) within the funding timeframe (13) (see **Figure 2**).

All practitioners in the trial (n=41) were invited to complete Costs Diaries to record their time and costs involved in implementing and delivering FT. 26 practitioners (63%) responded detailing the costs involved in delivering FT to 50 families. They were typically aged in their early forties (Mn=40.6; SD= 10.2), had worked as practitioners for an average of 15 years (SD = 6.7) and in 80% of cases (22/26) had experience of working across multiple settings (e.g., AMHS, CAMHS, and child protection services). Most were employed as social workers or social care workers (20/26), with a smaller number of clinical nurse specialists (2/26) and psychologists (4/26). Almost three fifths (15/26) worked in AMHS, approximately one third (9/26) in CAMHS and the remaining fifth in either primary care or the national child protection agency (Tusla) (5/26).

### Randomisation, allocation concealment, and blinding

Families were blindly randomised within each site area on a 2:1 basis to FT or to a services-as-usual, wait-list control group. This ratio, while it leads to a small reduction in statistical power, is ethically more desirable as it allows for the inclusion of a larger intervention group. Randomisation and allocation took place following family recruitment and baseline assessment, and was conducted by an independent consultant (unconnected to recruitment, data collection, or data analysis), using the SNOSE (Sequentially Numbered Opaque Sealed Envelopes) method (34). The independent consultant privately informed practitioners of the family’s group allocation, and concealed the randomisation and allocation sequence from the research team. Due to the nature of the intervention, neither family participants nor practitioners were blind to allocation. However, the researchers involved in data collection and statistical analysis were blind to treatment allocation. Participants and practitioners were requested not to disclose their group allocation to the research team at the follow-up assessment. To limit contamination between the intervention and control groups, practitioners who delivered FT were not involved in delivering services to the control group (and vice versa).

### Procedure

The trial received ethical approval from Maynooth University, as well as from an additional three ethics committees linked to collaborating organisations, including the Health Services Executive Research Ethics Committee, Tusla Ethics Review Committee, and the Saint John of God’s Research Ethics Committee. Families (parents and children 5–18 years) were recruited by practitioners in each site from their existing waiting lists. Sites had a prior installation/implementation period in order to allow practitioners to train and gain experience in delivering FT, and each site also had a designated lead person responsible for promoting referrals to FT and the RCT. Recruitment brochures and posters were designed by the research team in collaboration with a number of site personnel and used to inform families and practitioners about FT and the study. Recruitment commenced in March 2019 and was carried out by referring practitioners on a staggered basis. Once practitioners assessed the suitability of the family for FT and the RCT, and secured consent from parents for their contact details to be passed in confidence to the research team, parents were then contacted by the fieldwork coordinator via telephone to arrange for one of the research team to visit them to explain the research. Researchers met with parents in the family home, or, if preferred, in a local family/health care centre. At each data collection point (T0 and T1), families were given information sheets and their written informed consent was obtained. Data were collected from one parent only (71 mothers, 12 fathers). Parent informants all had a formal/working diagnosis and were in treatment for mental illness. For measures of child functioning, the parent had to select a child aged 5 to18 years old to report upon. Families were provided with a small ‘thank you’ for their participation in the research, in the form of a shopping voucher worth €20 at each data collection visit. Practitioners informed families of their allocation within two weeks of the baseline assessment, and arranged a suitable time for FT sessions.

Prior to the COVID-19 pandemic in mid- March 2020, the follow-up assessment (T1) took place six months after the baseline assessment (T0). However, due to intermittent periods of COVID-19 public safety restrictions in 2020-2021, both delivery of the intervention and data collection had to be paused for 4-6 months (i.e. from mid-March to July 2020, November 2020, and January 2021). Therefore, assessments affected by the restrictions were collected 4-6 months later than originally planned. Taking the pause into account, we still considered the follow-up assessment time for these families to also be at 6 months. We compared outcomes in the analysis for assessments conducted before and after the COVID-19 restrictions. It was not possible to conduct the planned 12-month assessment within the funding timeframe. Due to the lockdowns, recruitment was extended to April 2021 and data collection ended in December 2021.

### Measures

A ‘Profile Questionnaire’ was developed specifically for purposes of the study in order to elicit demographic and background information on participating families. This provided important data for describing participant characteristics, testing the equivalency of the control and intervention groups, and conducting attrition analyses. A number of psychometrically robust, parent-report measures were also administered to assess primary and secondary outcomes as described below.

### Primary outcomes

Our two primary outcomes were family functioning and child psychosocial functioning, both of which represent key objectives of the FT programme (19). *Family functioning* was assessed with the Systematic Clinical Outcome and Routine Evaluation (SCORE-15), a 15-item, reliable and validated parent-report measure of family communication, relationships, and functioning (35). The SCORE-15 has three dimensions or subscales including: ‘Strengths and adaptability’; ‘Overwhelmed by difficulty’; and ‘Disrupted communication’, with lower scores on each indicating better family functioning. The clinical cut-off score is 39 for adults, with population norms of 26.

*Child psychosocial functioning* was measured by the 25-item Strengths and Difficulties Questionnaire (SDQ) (36), a parent-report, psychometrically sound questionnaire designed to assess child conduct problems, hyperactivity, emotional symptoms, peer problems, and pro-social behaviour for 3–18 year-olds. Higher scores indicate more emotional and behavioural difficulties. The clinical cut-off point is 17, with a borderline score of 14.

The following secondary outcomes were also assessed.

- *Child depression* was assessed using the 10-item, psychometrically robust, parent-report, ‘Major Depression’ subscale from the Revised Children’s Anxiety and Depression Scale (RCADS) (37). Higher scores indicate more severe child depression. The clinical cut-off score is 10, with a borderline score of 8.
- *Child anxiety* was assessed using the 5-item version of the Screen for Child Anxiety Related Disorders (SCARED-5) (38). The SCARED-5 measures generalised anxiety disorder, panic/somatic, separation anxiety, social phobia, and school phobia. Higher scores indicate more anxiety, with a clinical cut-off score of 3.
- *Parental mental health* was assessed with the BASIS-24 (Behaviour and Symptom Identification Scale 24), a 24-item, parent-report questionnaire of mental health functioning in clinical populations (aged > 18) across six major areas: depression/functioning, relationships, self-harm, emotional lability, psychosis, and substance abuse (39). Higher scores indicate worse functioning. The clinical cut-off score is 35.
- *Parental coping and resilience* was measured with the Coping Self-Efficacy questionnaire (CSE), a 26-item, parent-report measure of parental confidence in performing coping behaviours when faced with life challenges (i.e. use of problem-focused coping, ability to stop unpleasant emotions and thoughts, receipt of support from friends and family). Higher scores indicate better coping ability, with a normative mean score of 137.4 (SD=45.6) (40).
- *Parental understanding of mental illness* was assessed using the Parental Understanding of Mental Illness questionnaire (PUMI), a 20-item, parent-report, questionnaire devised by the research team in the absence of any psychometrically robust and validated measures to assess a parent’s understanding and experience of how their mental health affects their children. A key proximal objective of FT is to improve knowledge and understanding of parental mental illness amongst family members. Therefore, the PUMI assessed parental mental health literacy, their experience of living with mental illness and relationships with children, and their perceived level of family, social, and service supports. Higher scores indicate better mental health literacy.
- It should be noted that in the protocol we indicated that we would administer the Warwick-Edinburgh Mental Well-being Scale to assess partner mental health (13). While all parents were requested to ask their partner to complete this questionnaire, we had a very poor response rate (<10%) and these data, therefore, are not reported here. The reasons for the low response rate are unclear. It could be because the partner forgot or did not provide consent. It should also be noted, however, that over 40% of parents in the study did not cohabit with a partner.

### Costs measure

The costs of the intervention were assessed using an online, anonymous Cost Diary which was completed by practitioners who had delivered FT to intervention group families. The diary was used by practitioners to record their salary rate and to document their time (hours) spent on a range of activities, including training in FT, securing buy-in from management/colleagues and setting up referral structures, recruiting and engaging families in sessions, participating in peer supervision, travel, and other costs incurred by them during the delivery period (e.g. materials, travel). All provided their written informed consent to take part in this element of the study. Due to the delays caused by the COVID-19 restrictions, it was not possible to conduct the planned cost effectiveness analysis.

### Power analysis

A power analysis was conducted using the two primary outcome measures— SCORE-15 and the SDQ—in order to determine samples sizes sufficient to register significant change (13). For the outcome of family functioning (SCORE-15), we conducted a G*Power *t*-test calculation for the difference between two independent means (two groups). Based on previous SCORE-15 studies (41), and assuming *α* = .05, 80% power, two tailed testing, 15% attrition, and 2:1 allocation, a sample size of 144 participants was recommended (FT = 96, TAU = 48) to detect a change of 0.5. Similarly, for the outcome of child functioning (SDQ), G*Power *t*-test calculations were conducted for the difference between two independent means. Based on previous evaluations of FT using the SDQ (23, 25), and assuming *α* = .05, 80% power, two tailed testing, 15% attrition, and 2:1 allocation, a sample size of 38 participants (FT = 25, TAU = 13) would be required to detect a change between 0.7 and 1. As noted above, the COVID-19 restrictions severely curtailed our rate of recruitment.

### Intervention

FT is a manualised, strengths-based, 7-session, weekly programme for families where one or both parents have a mental illness. The programme/intervention is based on psycho-education, narrative, and systemic therapy and is designed to promote family understanding and communication about mental illness, reduce stigma, validate the perspective of each family member, identify individual and family strengths, and promote family relationships (e.g. mutual empathy and support), problem solving, care planning, resilience and utilisation of social supports (19). It is important to note that, similar to other evaluations (18–20), FT in the current study was delivered alongside services as usual, as outlined in the ‘Control group’ section below. Thus, Family Talk (or other whole-family programmes) is not considered a replacement for other types of mental health interventions. These whole-family programmes are specifically targeted at enhancing family communication and outcomes with regard to the impact of parental mental illness on child/family wellbeing (9–12).

FT uses an individual family format whereby the trained practitioner meets with parents and the children (and extended family members—e.g. grandparent—if requested by the parents). Children must be aged 5 years and over as FT is targeted at children who are able to verbally express their experiences. The first two sessions involve the practitioner and parent(s) and include a discussion of the family’s experience of mental illness while providing psychoeducation if required. In session three, the practitioner meets with each child alone to conduct an assessment and to identify any questions which the child(ren) may have in relation to their parent’s mental illness. Next, a planning meeting between the practitioner and parents is held, after which a whole-family session is organised to support family discussion and provide information on mental disorders as required. The intervention concludes with a follow-up meeting to check in and support the family going forward (**Figure 1**). Each session lasts 60–90 minutes. More detail on FT sessions and programme theory can be sourced in Beardslee’s studies (14, 18–21) and in the freely available online training in FT (www.emergingminds.com.au). In addition, more information on the key facilitative and inhibitive factors to implementing FT can be seen in our qualitative studies (5, 11, 12).

### Control group

Families assigned to the control group received services as usual which normally comprised medication, psychotherapy, and/or group intervention (e.g. Dialectical Behaviour Therapy, Stress Control) from their psychiatrist or another member of the Multi-disciplinary Team (MDT), or from their GP in line with usual practice. Therefore there was considerable heterogeneity in the nature of the supports provided to the treatment-as-usual control group across sites. Practitioners who delivered usual services were based in the same adult or child site as FT facilitators and similar to FT facilitators, were employed as social workers, social carers and psychologists. To limit contamination between the intervention and control groups, practitioners who delivered services to the control group were not involved in delivering FT to families. All control group families were offered FT following the T1 follow-up assessment.

### Treatment integrity and fidelity

For practitioners to be eligible to deliver FT as part of the trial, they were required to have at least three years’ experience in working with adult or child mental health and/or child welfare and protection services. On average, practitioners had relevant experience of 15 years (SD = 6.1), with three quarters having worked in multiple settings (e.g., AMHS, CAMHS, and child protection services). In addition, all clinicians were required to complete the certificate in online training in FT (www.emergingminds.com.au) and have received regular (at least monthly) supervision in FT delivery. Supervision sessions were 1-1.5 hours, coordinated by the designated lead person in each site, and provided peer support and guidance in FT training, engaging and working with families, securing buy-in from management and colleagues, setting up referral structures, and recruitment for the RCT. Consistency and quality of programme delivery were also promoted through the manualisation of the programme and completion of weekly session checklists, of which 90%+ was covered when participants completed the course. Families were judged to have completed the programme if they attended at least 7 sessions that included parent, child and family sessions.

The average hours delivered to intervention families was 10.14 (SD=5.85) but FT duration varied considerably between those who attended before or after the pandemic. Most families (76%) attended FT before the onset of the pandemic. While FT is a 7-session, weekly programme, average intervention duration for this group of families was 9.2 weeks (SD=2.15), taking into account the number of individual child sessions required and missing appointments due to illness or family crisis. In addition, in approximately one third of these families (31%), clinicians indicated they had provided an additional parent or child session where complex issues were raised.

Treatment fidelity was a substantial challenge for approximately one quarter (24%) of intervention families due, in large part, to delays/disruptions caused by the COVID-19 restrictions. In some cases (n=5), practitioners adapted FT using online platforms, which facilitated sessions with parents and older adolescents, but were not considered suitable for younger children or family sessions, and therefore completion of FT with these families was delayed for several months, meaning that delivery was disjointed. Some families (n=5) withdrew from the programme due to the COVID-19 disruptions. In addition, a small number of families (n=3) were not offered FT, due to staff shortages, discharges and a change in service priorities as a result of the COVID-19 restrictions. Therefore intervention duration for this group ranged from 0-3 sessions up to disjointed sessions delivered over a 4-6 month period.

### Attendance

Mean attendance in the intervention group (as recorded by practitioners) was 5.4 sessions (SD = 1.2), with 63% attending all sessions. This compares unfavourably with 80-90% attendance rates reported in the Beardslee studies (14, 18, 20). Mean attendance in other FT studies is generally not reported. Our qualitative analyses of family and practitioner experiences of FT indicated that the primary reasons for disengagement were mainly related to disillusionment due to delays/disruption caused by COVID-19, a family crisis, and a relapse in symptoms. Only two families highlighted parental stigma as a reason for disengagement (11, 12).

### Analysis

Data were analysed according to the Consolidated Standards of Reporting Trials (CONSORT) guidelines for RCTs, and in line with the CONSERVE-CONSORT checklist for RCTs conducted during the COVID-19 pandemic (42, 43). Considerable time and effort were invested in data cleaning (using Microsoft Excel VBA code) before analysis was conducted. Descriptive statistics were calculated and reported with means, standard deviations (SDs) and frequencies, with count data compared using Fisher’s Exact Test [F] for 2x2 tables or the Fisher-Freeman-Halton Exact Test (F) for larger tables. Continuous data were compared via independent samples t-tests. Normality assumptions of parametric tests were not violated. Between-group outcome results were calculated and reported with means, SDs, p and F values, eta squared and Cohen’s d effect sizes, whereby an effect size of 0.2 denotes a small effect, 0.5 a medium effect, and 0.8 a large effect of the intervention, as reported from SPSS v28.0 (44). The unit of analysis was the parent-child dyad where the parent selected a child to report upon.

It should be noted that the plan to analyse the data as repeated measures (13) was subsequently changed to Mixed Modelling (MM), treating baseline values as covariates. This decision was made before any data analysis was conducted (i.e. it was not made following results from an MMRM analysis). This is important in the context of researcher degrees of freedom and p-hacking (45). The advantage of this change meant that results could be interpreted across different baseline values, a fact that could be relevant for clinical practice. In addition, MM has the advantage that missing values need not be imputed in any way. We also changed the number of covariates in the model. Originally, we planned to control for baseline parent mental illness as a covariate that could interact with treatment allocation (13). However, further investigation of the literature indicated a likelihood that social disadvantage and partner mental health may potentially act as covariates (2, 25). Therefore, following discussion with the statistician (CM) and before any analysis was conducted, we decided that the analytic model would be enhanced if we added social disadvantage and partner mental health as covariates, in addition to the covariate of baseline psychopathology. The modelling was conducted based on fixed effects only, and Maximum Likelihood was used as the solution method as recommended by Field (2017) for fixed effects modelling (46). Analysis followed the intention-to-treat principle whereby data were included from all contactable participants regardless of programme attendance. A parallel per-protocol analysis excluded participants who did not complete the programme as intended by randomisation, i.e. attending at least 7 sessions that included parent, child and family sessions. Attrition analyses were carried out to assess differences in participant characteristics between those lost to follow-up and those who stayed in the trial.

As outlined earlier, the extended COVID-19 restrictions prevented us conducting the planned 12-month follow-up assessment within the funding timeframe; therefore, data were only available at baseline (T0) and at 6-month follow up (T1). Assessments affected by the COVID-19 suspensions were compared with data collected before the pandemic: 55% of 6-month data was due to be collected after mid- March 2020, with double the rate of attrition at 6-month follow up following COVID-19 (23% vs. 45%).

Given the COVID-19 disruption, it was not possible for us to conduct the planned cost-effectiveness analysis within the funding timeframe (13). Instead, as outlined earlier, we used the data collected in the practitioner Cost Diaries to calculate the per-family cost of delivering the intervention (including and excluding one-off, non-recurring costs). This enabled us to provide an indication of the approximate costs involved in preparing for, and delivering the intervention, as well as the approximate proportions of time spent on various activities, which should be useful for service planning in implementing FFP.

## Results

### Participant characteristics

Parent participants were predominantly female (86%), with a mean age of 40.5 years (SD = 6.81) (**Table 1**). Most parents, at baseline, had been diagnosed with anxiety/depression (57%), followed by Bipolar Disorder (20%), Borderline Personality Disorder (12%), PTSD (8%) and psychosis (2%). Nearly half (48%) were in their current episode for more than two years, 12% for 1-2 years, 14.5% for 6-12 months and 17% for< 6 months duration. The vast majority of parents (< 80%) were attending AMHS, with the remainder under the clinical care of their GP. Most of the index children were female (60%) with a mean age of 13.85 years (SD=4.44). More than half of children (53%) were attending CAMHS or a psychology/family support service. 76% of families were socially disadvantaged when compared with average Irish norms (47) (**Table 1**).

**Table 1:** Participant characteristics at baseline.

Participant characteristics	At baseline			Lost to follow up	
	Controls n = 28	Intervention n = 55	Between group comparison – p value/Effect size	Controls n = 20	Intervention n = 32
PMI gender (female/male)	26/2	44/10	.205^F^	18/2	24/7
PMI mean age (SD)	38.5 (6.51)	42.5 (7.10)	.019/*d* = 0.6	40.1 (6.80)	43.7 (7.39)
Mental illness -Anxiety/depression -Bipolar -BPD -Psychosis -PTSD	16 6 3 1 2	31 10 7 1 5	1^F^	12 4 3 0 1	20 7 3 1 1
Length of episode -< 6 months -6-12 months -1-2 years ->2 years	6 5 5 10	8 7 5 30	.321^F^	5 3 3 8	6 3 5 15
Child gender (female/male) (n=83)	16/12	34/21	.813^F^	11/9	20/12
Child mean age	12.2 (4.62)	14.5 (4.25)	.030/*d* = 0.5.	12.4 (5.00)	14.4 (4.01)
Child mental health -CAMHS -Other psychology/family service -No service	4 12 13	17 11 26	.090^F^	3 9 8	11 5 15
Family social disadvantage (Yes/No) ≥ 2/6 ^a^	24/4	38/17	.117^F^	17/3	21/11
Measure scores					
SCORE-15 total score	36.8 (10.48)	32.3 (11.34)	.085/*d* = 0.4	37.8 (9.76)	32.2 (9.89)
SDQ total score	13.0 (5.88)	13.3 (7.00)	.851/*d* = 0.0	13.9 (6.17)	14.7 (7.32)
PUMI total score	56.7 (7.07)	58.2 (6.48)	.325/*d* = 0.2	55.8 (7.65)	58.1 (6)
RCADS total score	6.8 (6.68)	6.2 (5.44)	.668/*d* = 0.1	6.8 (5.72)	5.8 (4.95)
SCARED total score	3.0 (2.60)	3.1 (2.38)	.911/*d* = 0.0	2.8 (2.59)	3.4 (2.48)
BASIS-24 total score	29.6 (14.95)	28.1 (14.14)	.644/*d* = 0.1	30.5 (14.85)	28.7 (14.75)
CSE total score	102.0 (51.63)	122.8 (51.46)	.092/*d* = 0.4	92.1 (44.6)	116 (47.28)

a Social disadvantage score. Families who scored two or more of the following six risk factors were socially disadvantaged compared to average social norms in Ireland: income below the current poverty threshold in Ireland, employment status, lone parent, parental education, large family, and ownership of residence (47). ^F^Freeman-Halton Exact. ^d^Cohen’s *d*.

Statistical analyses (Chi-square and two-sample t-tests) indicated no significant differences between intervention and control group participants with respect to baseline characteristics or measure scores, with the exception that control group parents and children were slightly younger (parents: 38.5 years [6.51] vs. 42.5 years [7.10]; children: 12.2 years [4.62] vs. 14.5 years [4.25]) (**Table 1**). No statistically significant differences in participant characteristics were found between those retained in the study and those lost to follow-up. While similar reasons for attrition were given across both groups (e.g. impact of COVID-19, family crisis, relapse), the higher rate of attrition from the intervention group (42% vs. 29%) may be related to family disengagement from the research process due to disillusionment in delays/disruptions in attending FT as a result of the pandemic restrictions (**Figure 2**).

### Intervention outcomes

**Figure 3:**
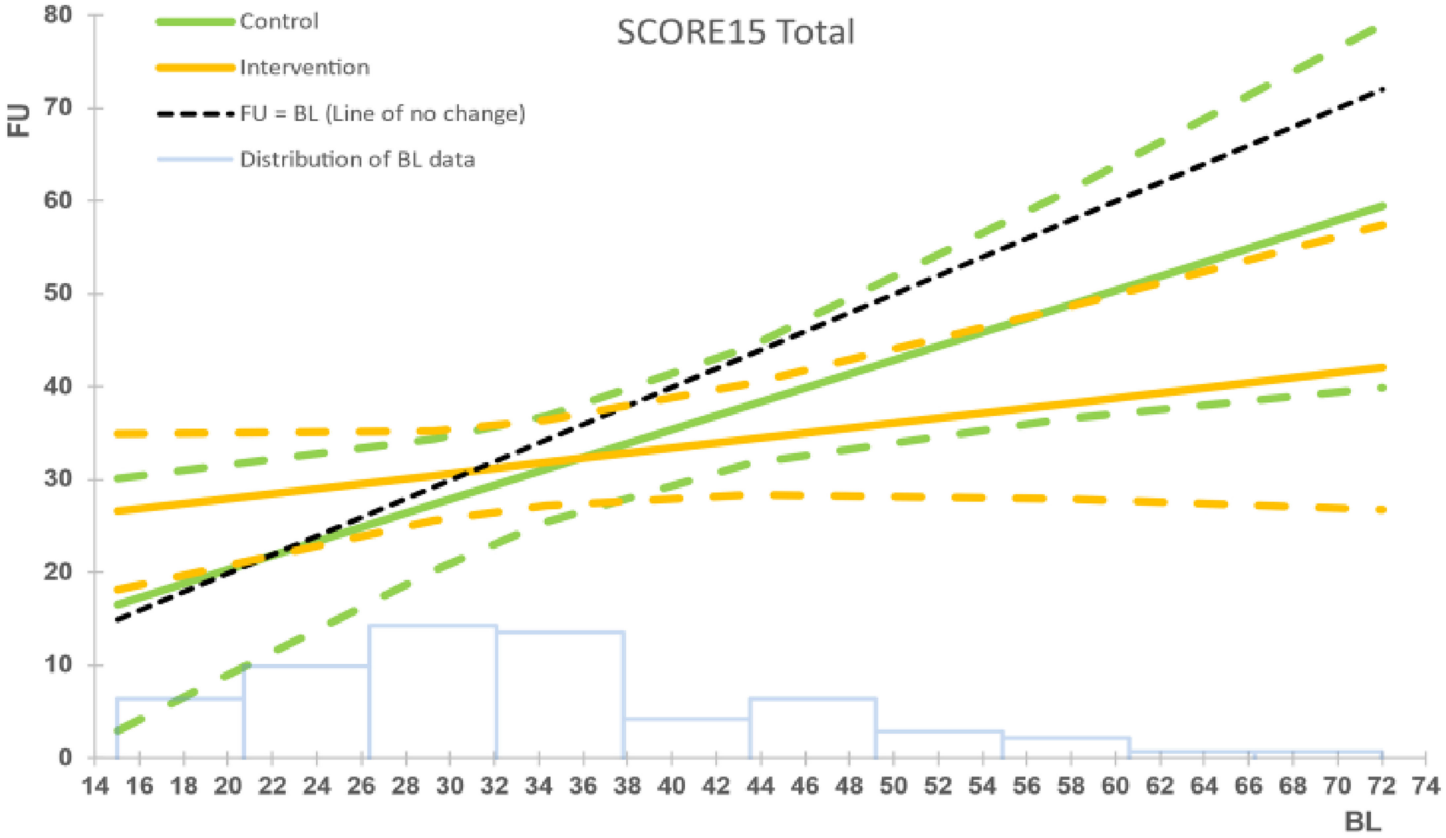
Intervention vs. control group for family functioning (SCORE-15), ITT analysis. The black dashed line represents the line of no change at follow up (FU) from baseline (BL). The solid coloured lines represent the model means for control (green) and intervention (orange) groups, with the corresponding dashed lines being the 95% confidence intervals. The faint blue histogram indicates the distribution of baseline data. The histogram is not to scale, and is intended to be purely suggestive. Bar height values must not be read from the chart.

**Figure 4:**
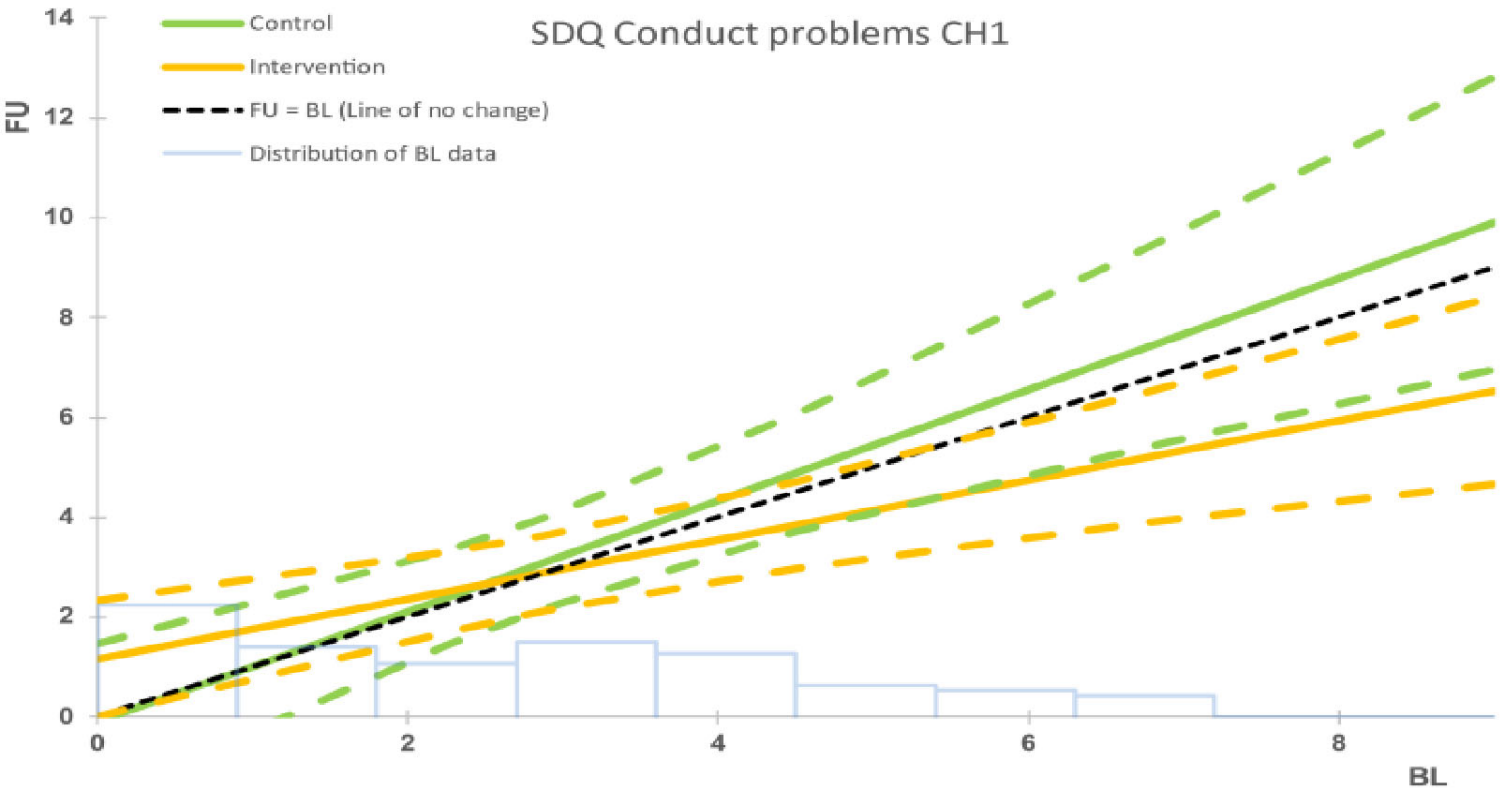
Intervention vs. control group for child behaviour (SDQ), ITT analysis. The black dashed line represents the line of no change at follow up (FU) from baseline (BL). The solid coloured lines represent the model means for control (green) and intervention (orange) groups, with the corresponding dashed lines being the 95% confidence intervals. The faint blue histogram indicates the distribution of baseline data. The histogram is not to scale, and is intended to be purely suggestive. Bar height values must not be read from the chart.

**Figure 5:**
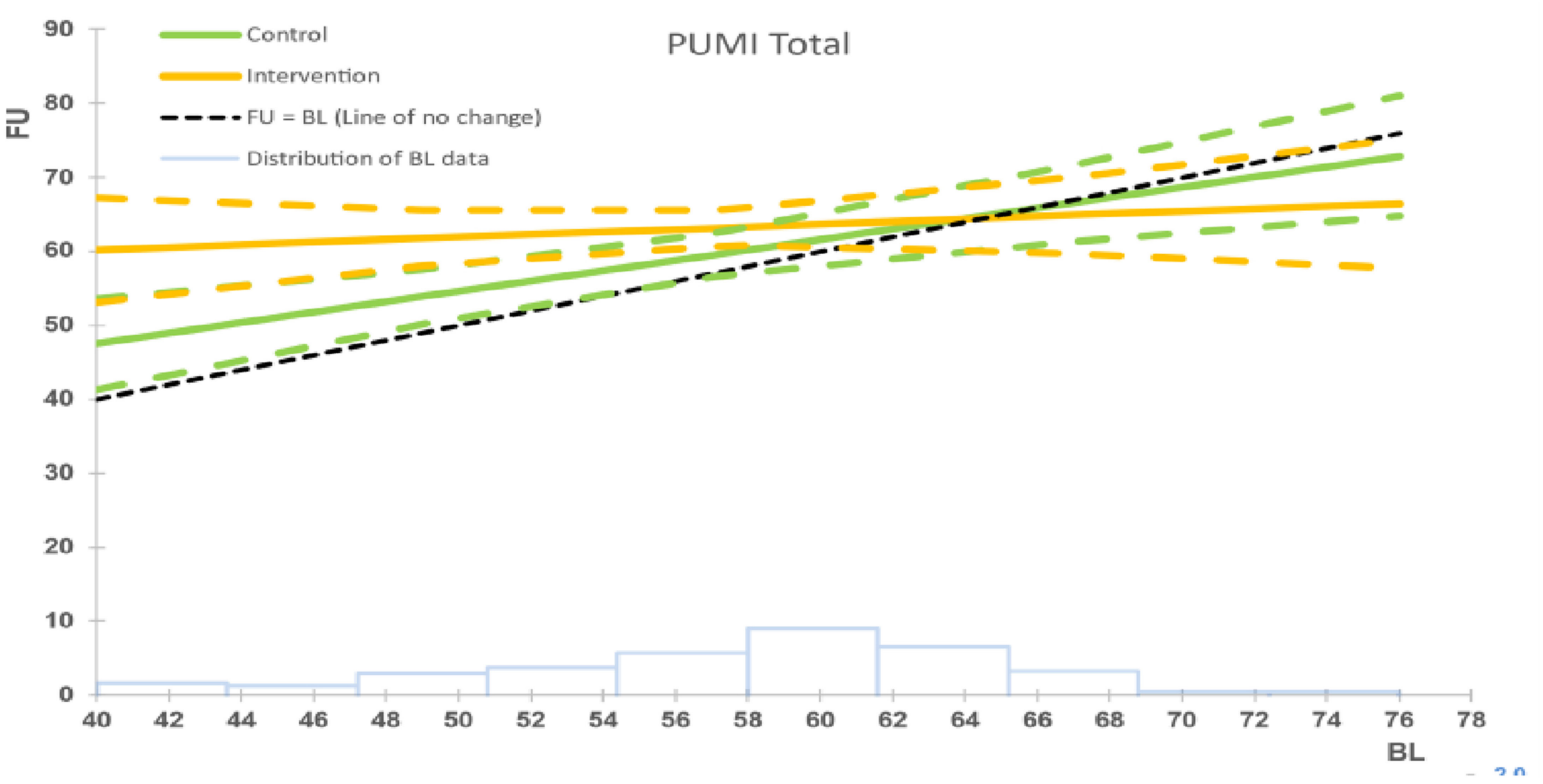
Intervention vs. control group for parental understanding of mental illness (PUMI), ITT analysis. The black dashed line represents the line of no change at follow up (FU) from baseline (BL). The solid coloured lines represent the model means for control (green) and intervention (orange) groups, with the corresponding dashed lines being the 95% confidence intervals. The faint blue histogram indicates the distribution of baseline data. The histogram is not to scale, and is intended to be purely suggestive. Bar height values must not be read from the chart.

The intention-to-treat (ITT) analyses revealed statistically significant between-group differences in two of the primary outcomes at 6-month follow up: family functioning (SCORE-15) and child behaviour (SDQ conduct scale), with both indicating medium effect sizes. There was also a statistically significant between-group difference in parental understanding of mental illness (PUMI total score), in that those who reported lower levels of mental-health literacy at baseline significantly improved at follow-up. No statistically significant between-group mean differences were found for the other outcomes, although positive trends favoured the intervention (**Table 2**, **Figures 3**–**5**). The per-protocol analyses yielded similar results (**Table 2**). Interestingly, while the SDQ total score (overall child psychosocial functioning) was not statistically significant in the main analysis, exploratory *post-hoc* testing across a range of baseline values in the per-protocol analysis indicated that children who reported baseline SDQ scores in the ‘borderline’ region (14.9-16) achieved statistically significant changes at follow up when compared to those with baseline scores in the ‘normal’ or ‘clinical’ regions (**Table 3**). We found no statistically significant differences between those whose attendance/assessments were delayed by the COVID-19 restrictions and those who attended/were assessed before the pandemic. Outcomes did not differ by type of mental illness. No harms were indicated either from the intervention or from the conduct of the RCT.

**Table 2:** Intervention outcome analyses.

	Intention to treat						Per protocol (control = 17, intervention = 27)	
	Raw data means (SD)				Allocation *F, p, d_s_*, $\eta_{p}^{2}$	Allocation * Baseline *F, p*, $\eta_{p}^{2}$		
	Control		Family Talk intervention					
	Baseline n=28	Follow-up n=20	Baseline n=55	Follow-up n=32			Allocation *F*, *p*, *d_s_*, $\eta_{p}^{2}$	Allocation * Baseline *F*, *p*, $\eta_{p}^{2}$
SCORE-15 total score ^1^	36.8 (10.48)	36.1 (14.13)	32.3 (11.34)	30.2 (11.10)	4.2,.045 *, 0.57, 0.08	2.2,.15, 0.04	4.5,.04 *, 0.64, 0.09	4.6,.037 *, 0.10
SDQ total score	13.0 (5.88)	14.8 (8.41)	13.3 (7.00)	13.5 (7.85)	2.3,.13, 0.42, 0.04	0.9,.36, 0.02	0.0,.84, 0.06, 0.00	0.1,.76, 0.00
SDQ conduct score	2.4 (1.77)	2.6 (2.85)	2.5 (2.23)	2.4 (2.18)	4.2,.046 *, 0.57, 0.07	3.8,.06, 0.07	2.3,.14, 0.46*, 0.05	5.6,.022 *, 0.11
SDQ emotional score	5.2 (2.98)	5.2 (3.22)	4.9 (2.92)	4.2 (2.76)	1.9,.17, 0.38, 0.04	0.6,.45, 0.01	1.1,.31, 0.31, 0.02	0.9,.34, 0.02
SDQ peer problems score	1.6 (1.89)	2.5 (1.40)	2.3 (2.18)	2.8 (2.14)	1.7,.19, 0.37, 0.03	0.1,.81, 0.00	0.5,.48, 0.21, 0.01	0.1,.77, 0.00
SDQ hyperactivity score	3.8 (2.76)	4.5 (3.36)	3.6 (2.79)	4.0 (3.21)	0.0,.87, 0.05, 0.00	0.4,.56, 0.01	0.7,.42, 0.24, 0.01	0.3,.60, 0.01
SDQ prosocial score	8.7 (1.61)	8.9 (1.23)	8.3 (2.12)	8.1 (2.20)	1.2,.29, 0.30, 0.02	0.2,.63, 0.00	0.6,.44, 0.24, 0.01	0.2,.63, 0.01
PUMI total score	56.7 (7.07)	57.7 (8.16)	58.2 (6.48)	63.9 (5.97)	3.7,.06, 0.54, 0.07	4.0,.050 *, 0.07	2.7,.11, 0.50, 0.06	2.4,.13, 0.05
RCADS depression score	6.8 (6.68)	7.2 (6.59)	6.2 (5.44)	6.2 (5.51)	1.2,.29, 0.30, 0.02	1.2,.27, 0.02	0.3,.61, 0.15, 0.01	0.0,.99, 0.00
SCARED total score	3.0 (2.60)	2.3 (1.66)	3.1 (2.38)	2.3 (1.76)	1.1,.29, 0.29, 0.02	0.6,.43, 0.01	2.6,.11, 0.49, 0.06	0.7,.40, 0.02
BASIS24 total score	29.6 (14.95)	28.0 (14.55)	28.1 (14.14)	23.9 (14.13)	2.3,.14, 0.42, 0.04	3.5,.07, 0.06	1.3,.27, 0.34, 0.03	2.2,.14, 0.05
CES total score	102.0 (51.63)	95.8 (53.73)	122.8 (51.46)	131.8 (52.33)	0.1,.74, 0.10, 0.00	0.9,.35, 0.02	0.1,.79, 0.08, 0.00	0.2,.66, 0.00

* p<.05 is statistically significant, highlighted in bold. All model follow-up mean results are evaluated at BASIS-24 = Mean (29.4). The F tests are all F (1, 52) except for CES and PUMI which are F (1, 49) and F (1, 51) respectively.

^1^ Clinical cut-off scores on measures: SCORE-15 – clinical 39, non-clinical 26; SDQ total score – clinical 17, borderline 8; RCADS depression score – clinical 10, borderline 8; SCARED – clinical 3; BASIS-24 – clinical 35; CES – mean 137.4, SD=45.6.

**Table 3:** SDQ total.

	Baseline value	Follow up Control Mean	Follow up Intervention Mean	FU Difference at given baseline value [95% CI], *p*	Cohen’s *d_s_* [95% CI]
**SDQ Total**	0.0	3.4	1.5	-1.9 [-9.3, 5.5],.61	0.16 [-0.45, 0.76]
	8.0	10.7	8.2	-2.5 [-6.7, 1.7],.24	0.36 [-0.25, 0.97]
	14.9 (BL mean)	16.9	14.0	-3.0* [-5.8, -0.1],.041*	0.63 [0.03, 1.24]
	16.0	18.0	15.0	-3.0* [-5.9, -0.1],.040*	0.64 [0.03, 1.24]
	24.0	25.3	21.7	-3.6 [-8.8, 1.6],.17	0.42 [-0.19, 1.03]
	32.0	32.3	28.4	-4.2 [-12.8, 4.4],.34	0.29 [-0.31, 0.90]

* p<.05 is statistically significant, highlighted in bold.

### Covariate analyses

In the ITT analyses, we found statistically significantly reduced child anxiety/depression (SCARED, RCADS) and improved parental mental health (BASIS 24) at 6-month follow up in higher functioning families (i.e. who were not socially disadvantaged and where a partner had good mental health, as reported by the parent with mental illness). Similarly, parents who reported less severe mental health symptoms at baseline reported significantly improved coping resilience (CSE) at follow-up. In the per-protocol analyses, further statistically significant benefits were found, with good partner mental health linked to improvements in a range of outcomes including: overall child wellbeing and behaviour (SDQ total score); child depression and anxiety (RCADS, SCARED); parental resilience (CSE); and parental understanding of mental illness (PUMI). In addition, significantly improved child depression and prosocial behaviour (RCADS, SDQ prosocial subscale) were seen in more socioeconomically advantaged families, while less severe PMI at baseline was related to significantly reduced child hyperactivity (as measured by the SDQ hyperactivity score) (see **Table 4**). Overall, families who completed FT had less severe mental illness and more partner and socioeconomic supports compared to those that dropped out after less than 3 sessions.

**Table 4:** Impact of covariates on outcomes.

Measure	Intention to treat			Per protocol		
	Partner mental health	Social disadvantage	BASIS 24 Total BL	Partner mental health	Social disadvantage	BASIS24 Total BL
SCORE15 total	F(3, 52) = .685, p=.565, $\eta_{p}^{2}$ = .04	F(1, 52) = 1.743, p=.193 d = .37, $\eta_{p}^{2}$ = .03	F(1, 52) = 2.324, p=.133 d = .42, $\eta_{p}^{2}$ = .04	F(3, 44) = .210, p=.889, $\eta_{p}^{2}$ = .01	F(1, 44) = .721,p= .401, d = .26, $\eta_{p}^{2}$ = .02	F(1, 44) = 3.265, p=.078, d = .54, $\eta_{p}^{2}$ = .07
SDQ total	F(3, 52) = 1.835, p=.152, $\eta_{p}^{2}$ = .10	F(1, 52) = .076, p=.784 d = .08, $\eta_{p}^{2}$ = .00	F(1, 52) = .209, p=.649, d = .13, $\eta_{p}^{2}$ = .00	F(3, 44) = 2.942, p=.043 *, $\eta_{p}^{2}$ =.17	F(1, 44) = 1.978, p=.167, d = .42, $\eta_{p}^{2}$ = .04	F(1, 44) = 1.504, p=.227, d = .37, $\eta_{p}^{2}$ = .03
SDQ emotional	F(3, 52) = 1.667, p=.186, $\eta_{p}^{2}$ = .09	F(1, 52) = .069, p=.794 d = .07, $\eta_{p}^{2}$ = .00	F(1, 52) = .448, p=.506 d = .19, $\eta_{p}^{2}$ = .01	F(3, 44) = 1.816, p=.158, $\eta_{p}^{2}$ = .11	F(1, 44) = .087, p=.770, d = .09, $\eta_{p}^{2}$ = .00	F(1, 44) = .551, p=.462, d = .22, $\eta_{p}^{2}$ = .01
SDQ peer problems	F(3, 52) = 1.522, p=.220, $\eta_{p}^{2}$ = .08	F(1, 52) = .092, p=.763 d = .08, $\eta_{p}^{2}$ = .00	F(1, 52) = 1.765, p=.190, d = .37, $\eta_{p}^{2}$ = .03	F(3, 44) = 1.935, p=.138, $\eta_{p}^{2}$ = .12	F(1, 44) = .002, p=.965, d = .01, $\eta_{p}^{2}$ = .00	F(1, 44) = 2.145,p= .150, d = .44, $\eta_{p}^{2}$ = .05
SDQ conduct	F(3, 52) = 2.533, p=.067, . $\eta_{p}^{2}$ . = .13	F(1, 52) = .447, p=.507 d = .19, $\eta_{p}^{2}$ = .01	F(1, 52) = .041, p=.840 d = .06, $\eta_{p}^{2}$ = .00	F(3, 44) = 2.868, p=.057, $\eta_{p}^{2}$ = .16	F(1, 44) = .007, p=.933, d = .03, $\eta_{p}^{2}$ = .00	F(1, 44) = .287, p=.595, d = .16, $\eta_{p}^{2}$ = .01
SDQ hyperactivity	F(3, 52) = 2.366, p=.082, $\eta_{p}^{2}$ = .12	F(1, 52) = .698, p= .407 d = .23, $\eta_{p}^{2}$ = .01	F(1, 52) = 3.780, p=.057 d = .54, $\eta_{p}^{2}$ = .07	F(3, 44) = 2.378, p=.083, $\eta_{p}^{2}$ = .14	F(1, 44) = 3.608, p=.064, d = .57, $\eta_{p}^{2}$ = .08	F(1, 44) = 5.221, **p=.027** *,d = .69, $\eta_{p}^{2}$ = .11
SDQ prosocial	F(3, 52) = 1.338, p=.272, . $\eta_{p}^{2}$ = .07	F(1, 52) = .451, p=.505 d = .19, $\eta_{p}^{2}$ = .01	F(1, 52) = 1.461, p= .232 d = .34, $\eta_{p}^{2}$ = .03	F(3, 44) = 5.034, p=.004 *, $\eta_{p}^{2}$ = .26	F(1, 44) = 6.127, **p=.017** *, d = .75, $\eta_{p}^{2}$ = .12	F(1, 44) = 6.306,**p= .016** *,d = .76, $\eta_{p}^{2}$ = .13
PUMI total	F(3, 51) = 3.821, p=.015 *, $\eta_{p}^{2}$ = .18	F(1, 51) = .001, p=.977 d = .01, $\eta_{p}^{2}$ = .00	F(1, 51) = 4.217, **p=.045** *d = .58, $\eta_{p}^{2}$ = .08	F(3, 43) = 4.435, p=.008 *, $\eta_{p}^{2}$ = .24	F(1, 43) = .643, p=.427, d = .24, $\eta_{p}^{2}$ = .01	F(1, 43) = 6.893, p=.012*, d = .80, $\eta_{p}^{2}$ = .14
RCADS total	F(3, 52) = 5.283, p=.003 *, $\eta_{p}^{2}$ = .23	F(1, 52) = 8.675, p=.005* d = .82, $\eta_{p}^{2}$ = .14	F(1, 52) = 1.877, p=.177 d = .38, $\eta_{p}^{2}$ = .03	F(3, 44) = 7.349, p<.001 *, $\eta_{p}^{2}$ = .33	F(1, 44) = 18.958, p<.001 *, d = 1.31, $\eta_{p}^{2}$ =.30	F(1, 44) = 3.988, p=.052, d = .60, $\eta_{p}^{2}$ = .08
SCARED total	F(3, 52) = 3.263, p= .029 *, $\eta_{p}^{2}$ = .16	F(1, 52) = .083, p=.774 d = .08, $\eta_{p}^{2}$ = .00	F(1, 52) = .159, p=.692 d = .11, $\eta_{p}^{2}$ = .00	F(3, 44) = 3.197, p=.032 *, $\eta_{p}^{2}$ = .18	F(1, 44) = .862, p=.358, d = .28, $\eta_{p}^{2}$ = .02	F(1, 44) = .527, p=.472, d = .22, $\eta_{p}^{2}$ = .01
BASIS24 total	F(3, 52) = .654, p=.584, $\eta_{p}^{2}$ = .04	F(1, 52) = 7.099, p=.010 *, d = .74, $\eta_{p}^{2}$ = .12		F(3, 44) = 1.211, p=.317, $\eta_{p}^{2}$ = .08	F(1, 44) = 1.327, p=.256, d = .35, $\eta_{p}^{2}$ = .03	
CES total	F(3, 49) = 2.624, p=.061, $\eta_{p}^{2}$ = .14	F(1, 49) = 1.487, p=.229 d = .35, $\eta_{p}^{2}$ = .03	F(1, 49) = 5.305, p=.026 *, d = .66, $\eta_{p}^{2}$ = .10	F(3, 41) = 7.360, p<.001 *, $\eta_{p}^{2}$ = .35	F(1, 41) = .104, p=.749, d = .10, $\eta_{p}^{2}$ = .00	F(1, 41) = 13.992, **p<.001** *,d = 1.17, $\eta_{p}^{2}$ = .25

*p<.05 is statistically significant, highlighted in bold.

### Implementation costs

As outlined earlier, data were collected from 26 practitioners who had delivered FT to 50 families. On average, practitioners’ hourly salary was €31.27 (SD=7.37) and they spent 45.85 mean hours (SD=6.33) in FT implementation and delivery, with 46% of their time involved in one-off, non-recurrent costs (i.e. training in FT, securing buy-in with management and colleagues), 34% in recruiting families and delivering FT sessions, and 20% in supervision. There were no significant differences in salary or hours according to type of site (AMHS, CAMHS, Primary Care). The overall cost of implementation and delivery was €761.50 per family, when one-off costs were included compared to €415.31 per family when only recurring costs were considered (see **Table 5**).

**Table 5:** Costs of implementing Family Talk.

Salary per hour (mean €, SD)	31.27 (7.37)
One off costs (mean hours, SD)	
-Family Talk training	10.86 (3.15)
-Securing buy-in^1^	10.43 (12.27)
*Subtotal*	*21.29 (7.71)*
Recurring costs (mean hours, SD)	
-Recruiting families	2.74 (3.34)
-FT sessions	10.14 (5.85)
-Travel to family home	2.65 (5.90)
-Supervision	9.03 (4.72)
*Subtotal*	*24.56 (4.95)*
Other recurring costs (mean €, SD)	
-Materials	6.96 (2.30)
-Travel for home visits	23.73 (6.16)
*Subtotal*	*30.69 (8.46)*
Total mean cost per clinician	1464.42 (124.89)
Total cost € of delivery to 50 families	38, 074.95
**Total cost € per family (including one-off costs)**	**€761.50**
**Total cost € per family (excluding one-off costs)**	**€415.31**

^1^ Buy-in involved meetings and presentations with management and colleagues to secure agreement to implement FT within their service as well as time involved in setting up referral structures.

The bold text merely highlights the most important bottom-line information - the overall costs of delivering the intervention, including and excluding one-off costs.

## Discussion

The findings from the current RCT indicate that, across different mental health settings and diagnoses, Family Talk (FT) led to improved family relationships and functioning and fewer child conduct problems and, where mental health literacy was lower at baseline, improved parental understanding of mental illness and its impact on children. There were additional improvements in the per-protocol analysis in overall emotional and behavioural functioning for children who scored in the borderline range on the SDQ at baseline. There were no statistically significant improvements within the main analyses (ITT or per protocol) for child depression, anxiety, or parental mental health symptoms although there were trends favouring FT. Interestingly, however, we found that parents with less severe mental illness at baseline, and families with more partner and socioeconomic supports, derived additional benefits from FT, including improvements in child depression/anxiety and prosocial behaviour and in parental mental health symptoms and resilience. This was particularly the case where families attended all sessions, underlining the importance of engagement and implementation fidelity for positive treatment outcomes (48).

Enhanced family functioning and communication is a key objective of FT (21) and research indicates that these may be important in protecting children from developing mental health problems (49). However, this outcome has not been commonly assessed within independent evaluations of FT. The current RCT included family functioning as a primary outcome in our protocol (13), and found that FT significantly improved family cohesion, communication and ability to deal with stresses. This is consistent with the positive changes in family functioning reported in studies conducted by Beardslee and colleagues (18, 20, 21) and also within a recently completed RCT conducted in Greece (24). In addition, we found significant improvements in the related, more proximal outcome of family understanding of, and communication about PMI within parents with lower levels of mental health literacy at baseline. Previous studies that assessed mental health literacy have similarly reported positive results (14, 18, 20, 23). Giannakopoulos et al. (2021) found that improved family functioning was associated with the greatest changes in children’s psychosocial outcomes (24), thereby suggesting that a focus on family relationships should be an important active ingredient in interventions for children of PMI. Interestingly, the relationship between family functioning and child outcomes in the current study is less clear as while there were improvements in child conduct at the overall group level, only higher functioning families with more socioeconomic supports reported benefits in a range of child internalising and externalising symptoms. Therefore, the participation of a high number of socially disadvantaged families in the current study (76%), as well as the impact of COVID-19 on attrition and family wellbeing, may have meant that positive trends favouring FT did not translate into statistical significance. Further mediator analyses are required to more fully investigate the relationship between intervention outcomes (e.g. child behaviour) and putative mechanisms of change (e.g. family functioning, parenting, parental mental health symptoms, parental readiness to engage).

Most previous RCT evaluations of FT have indicated improvements in child internalising and externalising symptoms (14, 18, 20, 24–26), although some mixed results have also been reported, with one study showing improvements in externalising but not in internalising symptoms (23), another finding improvements in neither (27), and a third indicating improvements in child but not parent report of child psychosocial functioning (21). Our study found improvements in child externalising (conduct) symptoms within the main ITT analysis, but only found improvements in overall child emotional and behavioural symptoms (SDQ total scale) for those who scored in the borderline region at baseline and whose families attended all sessions (per protocol analysis). Moreover, improvements in child depression/anxiety in the current study were also linked to families with better partner and economic supports, indicating that improvements in child internalising symptoms were only experienced by some subgroups. As above, it is possible that the high level of social disadvantage in the current study, along with the effect of COVID-19 on attrition and family wellbeing, may have undermined the impact of FT on child outcomes, although it must be kept in mind that previous studies indicate a range of positive and mixed results in this regard.

Likewise, we found improvements in parental mental health symptoms only for the higher functioning family subgroups that had more partner and economic supports, and/or reported less severe parental mental illness at baseline. A small number of earlier FT evaluations have reported improvements in parental mental health at the overall group level, albeit at 1.5 and 4.5 year follow up (20, 21, 24), so it is possible that a longer-term assessment would capture benefits for more parents in the current study that were not realised in the shorter term. Interestingly, there have been mixed results from previous studies that investigated the link between child outcomes and the severity of PMI. For example, two studies conducted in Finland and Sweden found no link between child outcomes and baseline severity of parental depression/change from baseline (25, 26) while, conversely, Giannakopoulos et al. (24) reported that improvements in depression in a sample of Greek parents, were associated with enhanced child psychosocial functioning. Therefore, the nature of the relationship between severity of PMI and child outcomes remains unclear. It is important to highlight that our results did not differ significantly by type of mental illness, which is similar to research undertaken by Pihkala et al. in Sweden (28–31), and has important implications for the roll out of the interventions across a range of diagnoses, although more research is required to investigate the effectiveness of FT for different mental disorders.

Little research, to date, has investigated the influence of socioeconomic status or partner mental health on intervention outcomes. A small number of previous studies of FT – two RCTs and a qualitative analysis – noted that disadvantaged families were more likely to disengage from FT (22, 23, 25). The findings reported here indicate that families with more socioeconomic and/or partner supports derived additional benefits with regard to improved child internalising symptoms (a secondary outcome). Moreover, we found that socially disadvantaged families, and particularly those without a supportive partner and with more enduring mental illness, were more likely to withdraw from FT due to family crises, relapse in symptoms, and stresses in daily living (11, 12). This pattern was exacerbated during the COVID-19 pandemic restrictions where such families reported particular struggles with mental health, and difficulties in managing child behaviour in the absence of external childcare and social supports (11, 12, 50). However, further research is needed to investigate the relative importance of these, and other variables, in influencing intervention effectiveness.

Therefore, it is likely that a continuum of higher and lower intensity interventions is required to meet the full spectrum of family needs. In the current study, offering additional FT sessions to families with complex needs appeared to improve their chances of deriving some benefits from the programme (in terms of the primary outcomes) (11, 12). Interventions on offer clearly differ across countries and regions. For example, in Finland and Sweden, FT is typically offered as part of the of the Effective Child and Family Programme following delivery of the less intensive, evidence-based ‘Let’s Talk’ programme where the practitioner meets initially with the service-user parent for 1-2 sessions (25). Family Options, delivered in the US, is an evidence-based, high intensity, 18-month intervention for parents with severe mental illness (17), but its effectiveness relative to FT has not yet been evaluated. Furthermore, in one mental health region in Ireland, FT is delivered as part of a family-focused initiative to support families with mental illness which involves delivery of a suite of evidence-based interventions, including Behavioural Family Therapy (10-14 sessions), Dialectical Behaviour Therapy (weekly sessions over two years) and Eolas (i.e. which provides separate peer-group programmes for service users and family/friends (8 sessions)) (51). However, this model has not yet undergone an impact evaluation. More research is required to identify which programmes work best for families with varying levels of mental illness, economic backgrounds and social supports.

The current study is the first to calculate the costs of implementing FT within routine mental health provision, and indicates that from a public healthcare perspective, FT is a low-cost intervention, and if positive outcomes can be maintained, may be cost-saving in the longer run given the typically high level of service utilisation among children of PMI (3, 9). This cost analysis should be helpful in informing future service planning by detailing the proportion of practitioner time dedicated to different activities (e.g. training, securing buy-in, setting up referral structures, family recruitment and delivery of sessions), which encouragingly demonstrates that 46% of costs included one-off, non-recurring costs. It should be noted, however, that the majority of FT facilitators were social workers and costs may vary across disciplines. In addition, the costs to families (e.g. travel) were not included. Furthermore, it is likely that a continuum of higher and lower intensity family-focused supports (with attendant cost implications) is required to meet the varying needs of families. For instance, we found that higher functioning families with more partner and economic supports derived more benefit from FT and that approximately one third of families presented with more complex needs and required additional sessions and supports. It should also be noted that the costs analysis included data from families that attended during the pandemic (24% of intervention families), with considerable variation in FT duration for this cohort, ranging from >3 sessions to some families requiring extra sessions to reorientate when there were disruptions in delivery over several months. Interestingly, average session hours did not differ much between those who attended before or during the pandemic (9.2 vs. 10.14 hours) but variation (SD) in sessions did (2.15 vs. 5.85 hours). Therefore, a degree of caution should be exercised in generalising costs to non-pandemic delivery. Issues related to stakeholder views on the installation and implementation of FT, fit with existing practice, sustainability and capacity to scale across health systems all have attendant cost implications and are discussed in greater detail in our accompanying qualitative analyses (5, 11, 12).

### Strengths and limitations

The current study is one of the largest RCTs of FT and the fourth independent evaluation performed internationally. In addition, it incorporated a usual services control group design, involved the delivery of the intervention within routine mental health settings, and across a range of mental health disorders, while it is also based on the assessment of important family, child and parent outcomes. It is also one of the first independent evaluations to include family functioning as a primary outcome and to investigate covariates of intervention effectiveness, highlighting that FT may work better for some subgroups than others. Furthermore, it is the first study to detail the costs associated with implementing FT (indeed such studies are scarce within the FFP field more generally), albeit it was not possible to conduct the planned cost-effectiveness analysis.

The limitations of the study were primarily related to the challenges posed by the COVID-19 pandemic restrictions during 2020-2021. Firstly, the rate of recruitment was severely impacted in this regard. Power to detect change may potentially have been affected as we found more significant change where more data were available. In addition, there were non-significant trends favouring the intervention on some outcomes which may possibly have converted to significance with a larger sample size. While other factors, such as staff shortages and family stigma also affected recruitment in the earlier stages of the research, we were broadly on target to recruit a much larger sample prior to the onset of the pandemic restrictions.

Secondly, the delivery of FT was delayed/disrupted for approximately one quarter of intervention families as a result of the restrictions. While a small number of practitioners continued to work with parents and older adolescents using online platforms during the pandemic, these were not considered suitable for child or family sessions, and in most cases FT delivery had to be suspended for several months, meaning that delivery was somewhat disjointed for these families. In addition, eight families withdrew from the service or had services withdrawn due to the impact of the pandemic. It is important to note, however, that most families (76%) received FT before the onset of the pandemic.

Thirdly, attrition from follow-up assessments doubled (45% vs. 23%) following the pandemic restrictions. The higher level of attrition in the intervention group compared to the control group (42% vs. 29%) appeared to be largely related to disillusionment with the delays and disruptions in FT delivery caused by the restrictions (11, 12). Interestingly, however, we found no significant differences in results between families whose attendance/assessments were delayed by the COVID-19 restrictions and those who attended or were assessed before the pandemic. Nevertheless, it is important to note that more than half (55%) of the 6-month follow-up data (and 10% of the baseline data) were collected during a time of severe pandemic restrictions when population mental distress was elevated due to isolation, financial stress and a lack of service/school/community supports (52–54), which likely impacted questionnaire responses, and possibly underestimated the positive impact of FT.

Furthermore, the restrictions meant that it was not possible to conduct the planned 12-month follow-up assessment within the funding timeframe. Previous evaluations of FT indicate that some improvements in parental mental health and child psychosocial functioning may only materialise in the longer term (21, 24). It was also not possible within the study timeframe, due to COVID-19 restrictions, to conduct the planned cost effectiveness analysis. Nonetheless, it is hoped that the current costs analysis, while rudimentary in nature, will help to inform future larger economic evaluations and encourage decision makers, managers and practitioners to consider incorporating low-cost FFPs into their service planning protocols.

A final limitation concerns the heterogeneity of interventions in the usual services control group, with a variety of medication, psychotherapy and group interventions delivered across mental health settings. It is possible that a more homogenous control group may have produced different results, but we cannot be sure to what extent this is likely without undertaking further research.

### Implications for practice, policy and research

The current study findings indicate that FT, a structured, whole-family intervention, is an effective and low cost intervention in improving family functioning, child behaviour and mental health literacy. The fact that families with better partner and socioeconomic supports reported additional benefits in child anxiety/depression and in parental mental health symptoms, highlights the importance of establishing a continuum of lower and higher-intensity service supports to meet the spectrum of family need. The study also provides evidence that FT can be successfully implemented with participants across different diagnoses and mental health settings (including interagency collaboration between adult and child services), thereby reflecting a “no wrong door” approach to identifying and supporting families. Families who attended all sessions also reported better results, underscoring the importance of engagement and implementation fidelity (48).

The longer-term sustainability of FFP in Ireland and elsewhere, requires a multi-level, public-health response that should include, for example: “think family” policy/practice standards; dedicated funding for FFP; managerial support to implement FFP; initiatives to reduce mental health stigma and recruitment barriers; and a continuum of FFP to broaden its capacity to identify and support families. “Think Family” policy/practice standards include: mandatory auditing of the parenting status of adult mental health users, as legislated in Norway; balancing the priority given to patient confidentiality with unmet family needs; increased collaboration between traditionally segregated AMHS and CAMHS services; and equipping clinicians with time and resources to undertake recruitment and delivery activities (52, 53). Evidence to date indicates that change in the provision of FFP is slow even in countries with mandatory reporting and that considerable time, resources and collective will are required to move away from the traditional biomedical and siloed approaches to treatment (33, 55).

Future research should focus on producing more large-scale, high quality, independent evaluations of FT across different cultural, policy, and mental health settings, and with a range of mental health diagnoses. These should also include, where possible, longer-term follow-up assessments and consideration of a range of outcomes relating to family functioning, child internalising and externalising symptoms, and parental wellbeing. The accompanying qualitative analyses also revealed benefits for sub-categories of parent and child wellbeing, including reduced stigma and feeling heard and validated, thereby highlighting outcomes that could be usefully assessed in future RCTs (11, 12). In addition, further research is required to investigate for whom the intervention works best and which variables most influence intervention effectiveness. While we explored the influence of several factors including baseline severity of parental mental illness, partner mental health and socioeconomic status, our qualitative analyses, and other studies, have identified a range of organisational, intervention and family factors that might be usefully tested as moderator and/or mediator variables in quantitative research; these include presence of a local champion, awareness-raising activities, adequate staffing, referral and supervision structures, treatment integrity, duration of delivery, family self –determination/readiness to engage, type and severity of mental illness, parenting and family functioning (11, 12, 24, 56–58). Larger-scale studies could model the influence and interaction of such variables. Lastly, more sophisticated cost-effectiveness and/or cost-benefit analyses are needed to inform the mainstream implementation of FFPs within routine mental health services.

## Conclusion

The findings from the current study demonstrate that even in a country that lacks a national “think family” policy/practice framework to support families with PMI, a low-cost, structured, whole-family intervention can be effective in improving family functioning and child behaviour. Additional benefits in child and parental mental health were noted for higher functioning families, indicating that a continuum of supports may be required to meet the many and often complex needs of families. The RCT (and the accompanying qualitative analyses reported elsewhere) demonstrate that FT can be successfully implemented with different mental health disorders and across a range of adult, child and primary care mental health settings. These findings are important in adding to the growing evidence base for FT, whilst also providing a robust basis to inform practice and policy development for families with PMI, both in Ireland and elsewhere. However, multi-level, public-health responses are required across jurisdictions, not only to promote the longer-term sustainability of FFP, but also to address the enduring political, cultural, organisational, and family barriers to change.

## Data availability statement

The raw data supporting the conclusions of this article will be made available by the authors, without undue reservation.

## Ethics statement

This randomised controlled trial involving human participants received ethical approval from the Social Research Ethics Committee in Maynooth University, Ireland (Reference number SRESC- 2018-100), as well as from an additional three ethics committees linked to collaborating organisations, namely, the Health Services Executive Research Ethics Committee, Tusla Ethics Review Committee, and the Saint John of God’s Research Ethics Committee. Parents/legal guardians provided written informed consent for themselves and their children to attend the Family Talk intervention. Parents/legal guardians provided written informed consent to provide data on family psychosocial functioning. Children did not provide data for the trial.

## Author contributions

MF: Conceptualization, Investigation, Methodology, Project administration, Software, Supervision, Writing – original draft, Writing – review & editing. CoM: Data curation, Formal analysis, Methodology, Software, Writing – review & editing. ChM: Conceptualization, Investigation, Software, Writing – review & editing. ShM: Data curation, Project administration, Writing – review & editing. SiM: Conceptualization, Funding acquisition, Investigation, Methodology, Project administration, Resources, Supervision, Writing – review & editing.
